# Role of the Global Fitness Regulator Genes on the Osmotic Tolerance Ability and Salinity Hazard Alleviation of *Trichoderma asperellum* GDFS 1009 for Sustainable Agriculture

**DOI:** 10.3390/jof8111176

**Published:** 2022-11-08

**Authors:** Valliappan Karuppiah, Xifen Zhang, Zhixiang Lu, Dazhi Hao, Jie Chen

**Affiliations:** 1 School of Agriculture and Biology, Shanghai Jiao Tong University, Shanghai 200240, China; 2 The State Key Laboratory of Microbial Metabolism, Shanghai Jiao Tong University, Shanghai 200240, China

**Keywords:** *Trichoderma asperellum*, velvet, salinity, cell wall integrity, ionic homeostasis, Na^+^ mitigation

## Abstract

Velvet family proteins are global regulators of fungal growth and development. Here, we reported the role of *Vel1* and *Lae1* from *T. asperellum* in osmotic tolerance. Deletion of the *Vel1* and *Lae1* genes led to the retardation of vegetative mycelial growth under saline conditions. The strain carrying the overexpression locus of the *Vel1* and *Lae1* genes was highly resistant to oxidative stress by upregulating the enzymes and genes involved in antioxidant activity. Major physiological changes in the cell wall and vacuoles occurred under high saline conditions. The *Vel1* and *Lae1* overexpression strains increased cell wall thickness and the number of vacuoles, which seems to lead to an increase of the osmolyte content of glycerol and proline. The absorption of Na^+^ content in the vacuole of the *Vel1* and *Lae1* overexpression strains was increased, while the absorption of Na^+^ was impaired in the *Vel1* and *Lae1* knock out strains, in which the Na^+^ was localized in the cell wall membrane. This result supported the significant correlation of the expression of genes with the ionic transportation in *T. asperellum*. Maize root colonization by the *Vel1* and *Lae1* gene overexpression strain was increased, which would mitigate the stress caused by the absorption of Na^+^ in the maize roots and increased the plant growth. Our results highlighted the importance of *Vel1* and *Lae1* proteins to the salinity stress tolerance of *T. asperellum* and the mitigation of Na^+^ stress to plants for sustainable agriculture.

## 1. Introduction

In nature, microorganisms in the soil are symbiotically associated with the plant’s rhizosphere [1]. The ecological relationships between plants and microbes exhibit a favorable effect on plant growth under different circumstances [2]. Among different abiotic stress conditions, salinity is a universal problem that affects plant growth in an irrigated field [3]. Under this circumstance, microbes play a vital role in mitigating saline stress in plants by augmenting water absorption capacity and nutrient uptake, thereby improving plant growth under salt stress [4]. *Trichoderma* is an important genus of fungus that has several beneficial effects upon plant growth and development [5]. *Trichoderma* colonize a broad spectrum of plant roots, including those of crop plants [6]. It is anticipated that *Trichoderma* have developed proficient schemes to overwhelm plant immunity and to create a suitable environment for growth and nutrient acquirement [7]. *Trichoderma* are renowned as biological control agents for the control of plant disease, growth and resistance against several abiotic stresses [8]. *Trichoderma* have also been used as an agent of bioremediation [9]. Plants treated with *Trichoderma* increase their growth and maintain the homeostasis of hormones needed to enrich salinity tolerance [6]. *Trichoderma* mitigates salinity stress and considerably enhances the physico-chemical characteristics of plants. *Trichoderma asperellum* has increased maize growth by mitigating NaCl [10]. Despite this data, the molecular mechanism of *Trichoderma* behind this salt resistance has not yet been determined. Until now, the mitogen-activated protein kinase (MAPK) signaling module has only been studied during the stress response. The most-researched MAPK component during salinity stress is the high osmolarity glycerol (HOG) pathway [11].

The velvet complex has been identified in numerous fungi. It is constituted by four genes, namely *VeA*, *VelB*, *VelC*, and *VosA* [12]. It plays an important role in protein interaction and DNA binding. These genes are involved in the various physiological functions, including growth and development, maintenance of fungal morphology, secondary metabolite production, and virulence [13]. In *Aspergillus nidulans*, *VeA* cooperates with *VelB* and *LaeA* to control fungal growth and the production of secondary metabolites [12]. In particular, *Vel*1 (orthologues to *VeA*) regulate the growth of fungi globally. In *Trichoderma*, *Vel*1 regulates chlamydospore formation and secondary metabolite production. In addition, *Vel*1 enhances secondary metabolite production, mycoparasitism, and the biocontrol capability of the *Trichoderma* [14]. This suggests that *Vel*1 is the global regulator of fungal growth and development. LaeA also regulates the process, including asexual reproduction and fruiting body development. The *Lae*1 of *Trichoderma reesei* (ortholog of *Aspergillus LaeA*) regulated the expression of carbohydrate-active enzymes (CAZymes) [15]. In this investigation, we have extended the hypothesis of global regulator with the goal of identifying the mechanism and importance of *Vel*1 and *Lae*1 on the salinity tolerance of *T. asperellum*, and therefore we analyzed the importance of the alleviation of salinity hazard by the *T. asperellum* GDFS 1009 Vel1 and Lae1 protein to maize growth.

## 2. Materials and Methods

### 2.1. Construction of Vel1 and Lae1 Knockout and Overexpression Strains

*Trichoderma asperellum* GDFS 1009 (CGMCC NO. 9512) was used as the wild-type fungus (TAWT) throughout the research. All fungi were cultivated in a petri dish by placing a 0.5 mm disc into the center of the potato dextrose agar and incubating it at 28 °C. Images were recorded by using a Shineso imaging system. Knockout of the *Vel*1 and *Lae*1 genes was carried out based on homologous recombination using the ATMT method as suggested by Karuppiah et al. [16] (Figure S1). In order to obtain the knockouts of *Vel1* and *Lae1* genes, 1.7 KB of *Vel1*- and 1050 bp of *Lae1*-coding regions in *T. asperellum* GDFS 1009 were replaced with 1.46 KB of hygromycin B phosphotransferase (hph). To this end, the upstream and downstream regions of the *Vel1* and *Lae1* genes were amplified using the primer pairs as shown in Table S1. The resulting PCR fragments of each *Vel1* upstream and downstream region were digested with HindIII/ SalI (5′) and BamHI/ SacI (3′), and the PCR fragments of the *Lae1* upstream and downstream regions were digested with HindIII/ KpnI (5′) and BamHI/ Xbal (3′) and ligated into the plasmid pC1300kh in order to form the knockout plasmids pC1300Vel1 and pC1300Lel1, respectively. Agrobacterium tumefaciens-mediated transformation (ATMT) was carried out to transfer the pC1300Vel1 and pC1300Lel1 into *T. asperellum* as described by Fu et al. (2012) [17]. The strains were purified by sub-culturing on PDA. The *Vel1* and *Lae1* gene knockouts of *T. asperellum* GDFS 1009 were named as Δ*Vel1* and Δ*lae1*, respectively. The *Lae1* overexpression segment containing the *TrpC* promoter, *Lae1* ORF, and the *TrpC* terminator cassette (Supplementary Figure S2) were cloned into pC1300N and transferred into *T. asperellum* GDFS 1009 using the ATMT method as suggested by Karuppiah et al. [16]. The *T. asperellum* GDFS 1009 *Vel1* overexpression strain used the same method. The *T. asperellum* GDFS 1009 strains containing the *Vel1* and *Lae1* gene overexpression cassette were named OE*Vel1* and OE*Lae1*, respectively. PCR and qRT-PCR were performed to confirm the knockout and overexpression of the *Vel1* and *Lae1* genes (Supplementary Figures S3 and S4) from the 20 positive clones. Further, the knockout and overexpression of the *Vel1* and *Lae1* gene strains were confirmed by morphological features, including the poor growth, loss of sporulation, and green-colored pigmentation in Δ*Vel1* and Δ*Lae1* compared to the wild type, while these morphological features were improved in the OE*Vel1* and OE*Lae1* strains (Supplementary Figure S5). The sequences of the *Vel1* and *Lae1* genes were submitted under the GenBank accession numbers MW419920 and OP753158, respectively.

### 2.2. Halotolerance Assay

The salinity tolerance of TAWT, OE*Vel1*, OE*Lae1*, Δ*Vel1*, and Δ*Lae1* were tested on potato dextrose agar (PDA), yeast extract molasses medium (YM), and Vogel’s minimal salt medium (VMS) supplemented with different concentrations of NaCl and KCl (0, 250 mM, 500 mM, 1 M, and 1.5 M). Strains were inoculated at the center of the petri dishes and incubated at 28 °C for five days. The radial growth of each strain was evaluated on the 3rd, 4th, and 5th day. For the determination of the spore formation, 10^6^/mL of spore suspension was inoculated into the YM broth supplemented with 0.5 M NaCl and 0.5 M KCl. After 7 days, the spores were counted using a hemocytometer.

### 2.3. Effect of Saline Soil and Water on the Growth of Two Mutant and Wild-Type Strains

Spore tolerance and survival were measured in water and soil amended with NaCl. The spores were inoculated into the water and soil containing 0.5 M NaCl and incubated at 28 °C for 5 days. The typical method of 10-fold dilution plating and a spore count conducted using a hemocytometer was used to count the spores of *Trichoderma*. 

### 2.4. Effect of Salinity on the Accumulation of Proline and Glycerol in the Wild-Type and Mutant Strains

The wild-type, knockout, and overexpression strains were cultured in a YM medium supplemented with or without 0.5 M NaCl at 180 rpm for five days at 28 °C. After growing, mycelia were harvested and powdered using liquid nitrogen. The powdered mycelia were subsequently used to measure the glycerol content using a glycerol assay kit (Chaoyan, Shanghai, China). For proline estimation, 0.5 g of the mycelium was homogenized using 3% aqueous sulfosalicylic acid and centrifuged for 10 min at 5000 rpm. Two hundred microliters of supernatant were mixed with 200 µL of glacial acetic acid and 200 µL of acid ninhydrin and incubated for 1 h. The reaction was stopped by placing the mixture in ice, and proline was separated using toluene and measured at 520 nm. The quantity of proline was calculated using the standard curve of proline using an regression equation.

### 2.5. Effect of Salinity on the Oxidative Stress Tolerance Ability of the Wild Type and Mutant Strains

The reactive oxygen species (ROS) produced during salinity stress were detected using the Active Oxygen ROS Detection Kit dichlorodihydrofluorescein diacetate (H_2_DCFDA), green, Keygen Biotech, Nanjing, China, according to the manufacturer’s instructions. In order to evaluate the vulnerability of the wild type and its mutants to oxidative stress, strains were cultured in YM medium supplemented with or without 0.5 M NaCl for 5 days at 28 °C. Superoxide dismutase (SOD) and catalase (CAT) activity were estimated using the Superoxide Dismutase Activity Assay Kit and Catalase Activity Assay Kit (Solarbio Lifesciences, Beijing, China), respectively, according to the manufacturer’s instructions. NADPH oxidase (NOX) activity was conducted as described by Montero–Barrientos et al. [18].

### 2.6. Quantitative Real-Time PCR Analyses

Expression of the genes related to oxidative stress (SOD, CAT, and NOX), osmolytes (glyceraldehyde dehydrogenase [GDH]), MAPK signal transduction (TMK2 and TMK3) and ion transportation gene (Na^+^ transporting ATPase ENA-1 (ENA-1), Na^+^/H^+^ exchanger (NHE1), cation H^+^ antiporter (CPA1), sodium calcium exchanger (NCX) and sodium proline symporter [PutP]) were studied in the *T. asperellum* WT, knockout (Δ*Vel1* and Δ*Lae1*), and overexpression *(*OE*Vel1* and OE*Lae1*) strains grown in the YM medium supplemented with or without 0.5 M NaCl for 4 days.

The total RNA was isolated using the FastPure Plant Total RNA Isolation Kit (VAZYME, Nanjing, China), and cDNA was synthesized using HiScript IIIRT Supermix for qPCR (+gDNA wiper) (VAZYME, Nanjing, China) according to the manufacturer’s instructions. Real-time PCR was carried out as suggested by Karuppiah et al. [16]. The primers used for the qRT-PCR are shown in Table S1. The expression fold was measured using the ΔΔC_T_ method relative to the WT grown under normal conditions. In order to normalize the gene expression, 18S rRNA was used. The results were the average of three independent biological replicates.

### 2.7. Effect of Salts in the Mutant and Wild-Type Strain on Cell Wall Integrity

Wild-type and mutant strains were cultured in a YM medium supplemented with or without 0.5 M NaCl at 180 rpm for 5 days at 28 °C. One mL of the culture was centrifuged, washed with the sterile PBS, and fixed with 2.5% glutaraldehyde for one hour. The samples were post fixed with 1% osmium tetroxide and dehydrated with acetone series embedded with epoxy and polymerized at 55 °C for 24 h. Ultrathin sections were coated onto the copper grid and observed under a biological transmission electron microscope (TEM) (Tecnai G2). The localization of Na and K inside different subcellular organelles was measured by averaging 20 cells using a field emission transmission electron microscope (EDS TEM) (TALOS F200X).

### 2.8. Root Colonization Assays under Both Normal and Saline Soil Conditions

For *Trichoderma* maize root colonization, maize seeds were surface sterilized with 70% ethanol and 10% hydrogen peroxide and then germinated in a petri dish as suggested by Morán–Diez et al. [19]. After germination in a petri dish, similarly sized seedlings were transferred to jars containing 1/2 strength Murashige and Skoog medium with vitamins and sucrose. The seedlings were grown under the controlled system suggested by Morán–Diez et al. [19]. After 5 days, 1% of the wild-type and mutant strains were inoculated into the jar, followed by the addition of 50 mM NaCL into the jar and further incubation for seven days. For root colonization, root samples were randomly selected from each group, stained using lactophenol cotton blue staining [20], and observed under a light microscope.

*Trichoderma* colonization on the maize root was determined through quantification of *Trichoderma* DNA in the roots using real-time PCR, as described by Poveda [21]. The DNA was extracted from the maize roots of each group using a Fastpure Plant DNA Isolation Mini Kit (VAZYME, Nanjing, China). Real-time PCR was performed using a 2× chemQ Universal SYBRqPCR Kit (VAZYME, Nanjing, China), and 10 ng of DNA was used to quantify the actin genes of *Trichoderma* and maize using standard curves in triplicate of five plant root samples. The quantity of *T. asperellum* DNA was evaluated relative to the quantity of maize DNA of each sample.

### 2.9. Sodium and Potassium Content Measurements Using Atomic Emission Spectrophotometry inside the Mycelium of Trichoderma and Plant Roots

Wild-type and mutant strains were cultured in YM medium supplemented with or without 0.5 M NaCl at 180 rpm for 5 days at 28 °C. The samples were centrifuged and washed with 10 mM MgCl_2_ before being acid extracted. The Na^+^ and K^+^ contents were estimated using ICP atomic emission spectrophotometry. The results are reported as the mean values of four experiments. *Trichoderma*-inoculated and non-inoculated maize plant root samples were washed in 10 mM MgCl_2_, dried, and acid extracted in order to estimate the Na^+^ and K^+^ contents using ICP atomic emission spectrophotometry (iCAP6300).

### 2.10. Statistical Analysis

All experiments were performed in triplicate and repeated for reproducible results. The graphs were constructed using Microsoft Office Excel and Origin 6.0 with standard error bars. The results shown in graphs were the mean ± SEM. For multiple comparisons, two-way ANOVA with post hoc LSD, Duncan, and Bonferroni corrections were performed using the SPSS 2.0. Student’s *t*-test was performed to check the significance of the gene expression between the different strains using the SPSS 2.0. *p* < 0.05 was taken as significant. The heat map was constructed using HemI. The principal component analysis was performed using the GraphPad Prism 9.0.

## 3. Results

### 3.1. Osmo-Tolerance Ability of Vel1 and Lae1 Protein

In this study, the role of the *Vel1* and *Lae1* genes on *T. asperellum* GDFS1009’s halotolerance property has been investigated. All of these strains grew well in 250 mM NaCl (Supplementary Figure S6). At 500 mM NaCl, the hyphal growth of all of these strains was reduced in VMS medium, whereas the growth of all of these strains was better in PD and YM agar (Supplementary Figure S6A,B). At a 1 M concentration of NaCl, the hyphal growth of OE*Vel1* and OE*Lae1* was reduced to 75% in PD and YM agar, whereas the Δ*Vel1* and Δ*Lae1* growth was reduced to 40%. Comparatively, OE*Vel1* and OE*Lae1* were better sustained in 1 M NaCl-supplemented VMS medium than the WT, Δ*Vel1*, and Δ*Lae1* strains (Supplementary Figure S6C). The highest colony growth of OE*Vel1* and OE*Lae1* in 1.5 M NaCl-supplemented YM agar demonstrated the osmo-tolerance ability of Vel1 and Lae1 proteins. The analysis of variance and principal component analysis showed that colony growth is highly dependent upon the NaCl concentration, the *Vel1*/*Lae1* gene, and the medium (Supplementary Figure S7).

### 3.2. The Vel1 and Lae1 Genes Increased T. asperellum Survival against Salinity Stress in Water and Soil

The results indicate that *T. asperellum* survives in saline water and soil (Supplementary Figure S8). The deletion of the *Vel1* or *Lae1* genes significantly increased the susceptibility of *T. asperellum* to saline water (*p*  <  0.05, Supplementary Figure S8D). The resistance of *T. asperellum* in saline water was increased by the overexpression of *Vel1* and *Lae1* genes. The soil saline resistance experiment also revealed a similar trend for the mutants (Supplementary Figure S8C).

### 3.3. Overexpression of Vel1 and Lae1 Gene Induced the Oxidative Stress Tolerance under Osmotic-Stress

**Figure 1:**
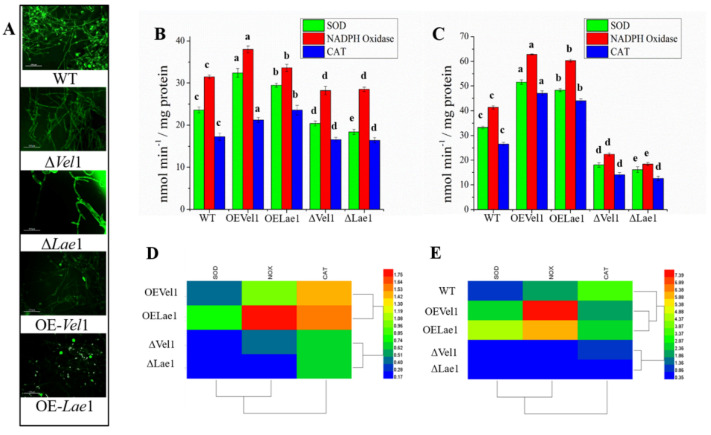
The reactive oxygen species production and oxidative stress response of *T. asperellum* WT, OE*Vel1*, OE*Lae1*, Δ*Vel1*, and Δ*Lae1* under salinity stress. (**A**) ROS production under saline stress. (**B**) Antioxidant activity under normal conditions. (**C**) Antioxidant activity under saline stress. (**D**) The relative expression of antioxidant genes (SOD, CAT, and NOX) under normal conditions. (**E**) The relative expression of antioxidant genes during saline stress (SOD, CAT, and NOX). Results were showed in terms of fold changes compared to the WT strains grown under normal conditions. Values are the average of biological triplicates. Error bars represent the standard error. Bars with different letters represent a statistically significant difference from each other at the level of *p* < 0.05 based on the ANOVA.

Salinity stress to *Trichoderma* increases the ROS, thereby interrupting redox homeostasis and resulting in oxidative damage. ROS production in the spores and mycelium of the WT, OE*Vel1*, OE*Lae1*, Δ*Vel1*, and Δ*Lae1* strains under saline conditions was detected using H_2_DCFDA under a fluorescence microscope. The results showed that the spores and mycelium of OE*Vel*1 and OE*Lae*1 produced less fluorescence than those of WT, Δ*Vel1*, and Δ*Lae1* (Figure 1A). These results suggest that the loss of the *Vel1* and *Lae1* genes leads to the increased production of ROS in spores and mycelia. The role of Vel1 and Lae1 proteins in the oxidative stress response have been studied by estimating the superoxide dismutase, NADPH oxidase, and catalase (Figure 1). The strains grown in 0.5 M NaCl significantly increased their accumulation of SOD activity in OE*Vel1*, OE*Lae1*, and WT (Figure 1B,C). Following the trend of superoxide dismutase, NADPH oxidase and catalase also significantly increased (*p* < 0.05) in OE*Vel1*, OE*Lae1*, and WT compared to Δ*Vel1* and Δ*Lae1*. The OE*Vel1*, OE*Lae1*, and WT strains grown under saline stress exhibited higher antioxidant activity than those from normal growth conditions (Figure 1B,C). In order to further investigate the effect of Vel1 and Lae1 proteins on the oxidative stress response during exposure to salinity stress, the transcription regulation of SOD, NOX, and CAT was explored using quantitative reverse transcription-PCR (qRT-PCR). As shown in Figure 1D,E, the highest transcript levels of NOX, CAT, and SOD were detected in the mycelia of OE*Vel1* (2.4-, 7.8-, and 2.1-fold, respectively) followed by OE*Lae1* (4.4-, 6.2-, and 2.7-fold, respectively), and WT (1.2-, 2.0-, and 3.8-fold, respectively) (Figure 1E). Δ*Vel1* and Δ*Lae1* resulted in the reduced expression of NOX, CAT, and SOD genes under saline conditions (Figure 1E). Among the WT, OE*Vel1*, OE*Lae1*, Δ*Vel1*, and Δ*Lae1* strains, significantly higher NOX1 transcript levels were detected in OE*Vel1*. Additionally, the gene expression detected under saline conditions was higher than that under normal conditions (Figure 1D,E). Thus, oxidative stress analysis revealed that the Vel1 and Lae1 proteins induce antioxidant activity to protect *T. asperellum* from oxidative stress generated during high salinity.

### 3.4. Vel1 and Lae1 Induced the Accumulation of Osmolytes and the MAPK Signaling Pathway under Salinity Conditions

**Figure 2:**
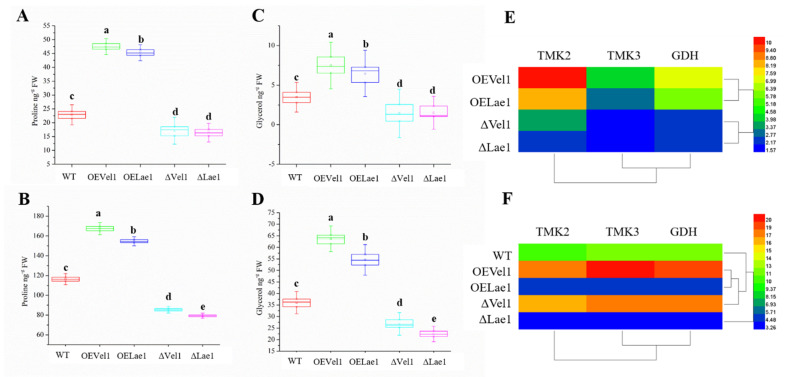
Osmolyte accumulation of *T. asperellum* WT, OE*Vel1*, OE*Lae1*, Δ*Vel1*, and Δ*Lae1* under salinity stress. Accumulation of proline under (**A**) normal and (**B**) saline conditions. Accumulation of glycerol under (**C**) normal and (**D**) saline conditions. The relative expression of MAPK signal transduction (TMK2 and TMK3) and GDH under (**E**) normal and (**F**) saline conditions as the fold ratio between the WT grown under normal conditions. Boxes with different letters represent a statistically significant difference from each other at the level of *p* < 0.05 based on ANOVA.

The accumulation of osmolytes, including proline and glycerol, was analyzed in all strains under normal and saline stress condition. Compared to normal conditions, saline stress conditions had significantly (*p* < 0.05) increased proline and glycerol contents (Figure 2A–D). The proline content of OE*Vel1* and OE*Lae1* differed significantly between Δ*Vel1* and Δ*Lae1* under both salts-treated and -untreated conditions (Figure 2A,B). This difference was generally due to the overexpression of the *Vel1* and *Lae1* genes. The proline content of all strains grown in 0.5 M NaCl-containing medium was significantly increased compared to the non-NaCl medium. The proline content of OE*Vel1* and OE*Lae1* was higher than the wild type (Figure 2B). The Δ*Vel1* and Δ*Lae1* strains showed less accumulation of proline compared to the wild type (Figure 2A,B). The pattern of glycerol content of the five strains was similar for both conditions (Figure 2C,D). The content of glycerol in the salt-treated *Trichoderma* was higher than that in the untreated *Trichoderma* (Figure 2D). OE*Vel1* and OE*Lae1* had significantly (*p* < 0.05) higher glycerol accumulation than wild type grown under saline conditions. Interestingly, the glycerol content was decreased in Δ*Vel1* and Δ*Lae1* under saline conditions (Figure 2D).

Furthermore, transcripts related to the metabolism of osmolytes and halo-tolerance were studied in WT, OE*Vel1*, OE*Lae1*, Δ*Vel1*, and Δ*Lae1* under both normal and saline conditions using qRT-PCR (Figure 2E,F). We observed that under saline stress (0.5 M NaCl), OE*Vel1* and OE*Lae1* strains showed a higher relative expression fold of glyceraldehyde 3-phosphate dehydrogenase compared to WT under the saline stress conditions, while the expression fold of glyceraldehyde 3-phosphate dehydrogenase was reduced in Δ*Vel1* and Δ*Lae1* compared to WT (Figure 2F). OE*Vel1* and OE*Lae1* upregulated the expression of TMK2 and TMK3 compared to WT grown without 0.5 M NaCl. OE*Vel1* and OE*Lae1* were up-regulated 18.36- and 16.54-fold compared to TMK2, respectively, under saline conditions. The TMK3 of OE*Vel1* and OE*Lae1* was upregulated 21.56- and 18.56-fold, respectively. Even though the expression of TMK2 (4.5- and 3.26-fold, respectively) and TMK3 (5.2- and 4.2-fold, respectively) were upregulated in Δ*Vel1* and Δ*Lae1*, the fold expression was lower than that of the WT, OE*Vel1*, and OE*Lae1* strains under osmotic stress conditions (Figure 2F).

### 3.5. Vel1 and Lae1 Induced the Expression of Ionic Transporter Genes Involved in the Na^+^ Homeostasis of T. asperellum

**Figure 3:**
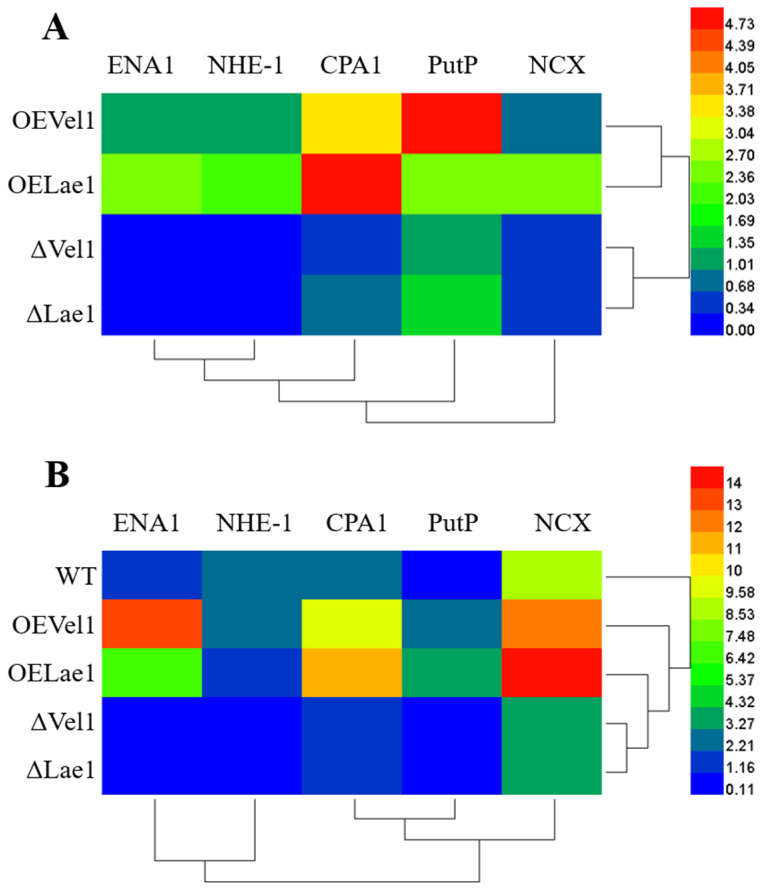
The regulation of putative Na^+^ transporter genes involved in Na^+^ homeostasis by the *Vel1* and *Lae1* genes under osmotic stress. The relative expression of putative Na^+^ transporter genes under (**A**) normal and (**B**) saline conditions as its fold ratio with that of the WT grown under normal conditions. Results are the average of five replicates for each treatment; the values given are the standard error of the mean.

In order to uncover the range of ionic transporter genes of *T. asperellum* that could be involved in the retention of Na^+^ homeostasis, BLASTP analysis was conducted using known sequences of other fungi. We recognized similar sequences in the *T. asperellum* CBS 433.97 genome. Sequence analysis showed that *T. asperellum* GDFS1009 had five genes encoding homologous proteins to fungal Na^+^-transporting ATPase ENA-1 (sodium p-type ENA-1), Na^+^/H^+^ exchanger, cation H^+^ antiporter, sodium–calcium exchanger, and sodium–proline symporter. These proteins were found to be involved in the saline tolerance of other fungi. Hence, we further investigated the expression of these genes in order to learn the link between the *Vel1* and *Lae1* genes and salinity tolerance. Higher fold changes were observed with the overexpression of the *Vel1* and *Lae1* genes compared to the WT grown under normal conditions (Figure 3A,B).

### 3.6. Vel1 and Lae1 Increased the Cell Wall Integrity and Absorption of Na^+^ under Osmotic Stress

**Figure 4:**
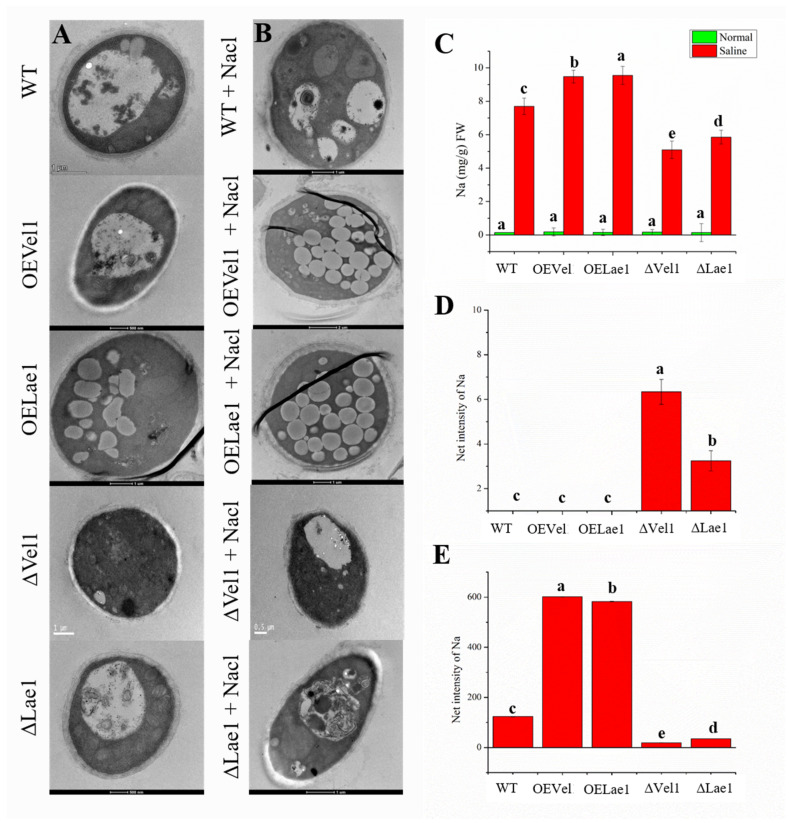
Osmo-adaptation of *T. asperellum* WT, OE*Vel1*, OE*Lae1*, Δ*Vel1*, and Δ*Lae1* under osmotic stress. (**A**) Transmission electron microscopic images of *T. asperellum* WT, OE*Vel1*, OE*Lae1*, Δ*vel1*, and Δ*lae1* under normal growth condition. (**B**) Changes in the cell wall thickness and number of vacuoles for *T. asperellum* WT, OE*Vel1*, OE*Lae1*, Δ*Vel1*, and Δ*Lae1* after exposure to hypo-osmotic stress. (**C**) Na^+^ absorption of *T. asperellum* WT, OE*Vel1*, OE*Lae1*, Δ*Vel1*, and Δ*Lae1* under normal and saline stress conditions. (**D**) Localization of Na^+^ in the vacuoles of *T. asperellum* WT, OE*Vel1*, OE*Lae1*, Δ*Vel1*, and Δ*Lae1* under saline stress conditions. (**E**) Localization of Na^+^ in the cell wall membrane of *T. asperellum* WT, OE*Vel1*, OE*Lae1*, Δ*Vel1*, and Δ*Lae1* under saline stress conditions. Values are the average of biological triplicates. Error bars represent the standard error. Bars with different letters represent a statistically significant difference from each other at the level of *p* < 0.05 based on the ANOVA.

We observed rapid changes in the cell surface due to salinity, and this could possibly be due to alteration of the cell wall morphology. Therefore, TEM was conducted in order to evaluate the roles of the *Vel1* and *Lae1* genes in the cell wall integrity of *T. asperellum*. The longitudinal and cross-sectional study of WT, OE*Vel1*, OE*Lae1*, Δ*Vel1*, and Δ*Lae1* hyphae exhibited alterations to the cell wall thickness, and it is dependent upon the strains being grown on normal or saline medium. Transmission electron microscopy images of WT, OE*Vel1*, OE*Lae1*, Δ*Vel1*, and Δ*Lae1* hyphae showed thin cell walls (<0.2 μm) (Figure 4A) under normal conditions. The cell walls of OE*Vel1* and OE*Lae1* were thickened (>0.2 μm) due to their growth in NaCl-containing medium, followed by the WT (Figure 4B). However, the cell walls of Δ*Vel1* and Δ*Lae1* were not thickened under saline conditions, though the rigidness of the Δ*Vel1* and Δ*Lae1* cell walls were decreased (Figure 4B). In addition, the cross sections of WT, OE*Vel1*, and OE*Lae1* hyphae grown under saline condition displayed rough surfaces of their cell walls due to the presence of extracellular polymeric substances (EPS). Subsequently, Δ*Vel1* and Δ*Lae1* grown under saline conditions showed smooth surfaces, which indicates the absence of EPS on the cell wall surface (Figure 4B). Further, the absorption of Na^+^ in WT, OE*Vel1*, OE*Lae1*, Δ*Vel1*, and Δ*Lae1* was estimated using ICP atomic absorption spectrophotometry (Figure 4C). OE*Vel1* and OE*Lae1* utilized significantly higher Na^+^ (*p* < 0.05) than the WT grown under saline conditions (Figure 4C). Δ*Vel1* and Δ*Lae1* absorbed significantly lower Na^+^ than the WT (Figure 4C).

Though WT, OE*Vel1*, and OE*Lae1* absorbed NaCl in order to tolerate the saline stress, NaCl in huge amounts might interrupt cellular activities under saline conditions. Therefore, the accumulation of NaCl in the subcellular organelles was scanned using EDS TEM. It has been widely accepted that Na^+^ is accumulated at higher concentrations in the vacuoles of OE*Vel1* and OE*Lae1* compared to the wild type (Figure 4D). Na^+^ was not detected in the vacuoles of Δ*Vel1* and Δ*Lae1*, while it was detected in the membrane of Δ*Vel1* and Δ*Lae*1 (Figure 4E). Thus, the *Vel1* and *Lae1* genes help *T. asperellum* to increase the accumulation of Na^+^ into the vacuoles in order to tolerate high salinity.

### 3.7. Vel1 and Lae1 Mitigate the Na^+^ during Co-Cultivation with Maize under Saline Conditions

Using qPCR, the colonization of *T*. *asperellum* WT, OE*Vel1*, OE*Lae1*, Δ*Vel1*, and Δ*Lae1* were calculated in maize roots under normal and saline conditions in a hydroponic system (Supplementary Figure S9). *T*. *asperellum* WT, OE*Vel1*, OE*Lae1*, Δ*Vel1*, and Δ*Lae1* colonization rates under both conditions are shown in Table 1 and Supplementary Figure S9. The DNA of *T*. *asperellum* WT, OE*Vel1*, OE*Lae1*, Δ*Vel1*, and Δ*Lae1* was detected in the roots of maize under both conditions. The ratio of plant:fungi DNA was increased in the roots of plants treated with OE*Vel1* and OE*Lae1* compared to the WT under both conditions (Table 1). The ratio of plant:fungi DNA in the roots of the plants treated with Δ*Vel1* and Δ*Lae1* was reduced compared to the WT under both conditions (Table 1). The ratio of plant:fungi DNA of OE*Vel1*- and OE*Lae1*-treated plants was higher under normal conditions compared to saline conditions (Table 1). These results correlated well with the microscopic image of the root colonization (Supplementary Figure S9).

The highest branching and thickening rates of the maize roots were observed in plants treated with OE*Vel1* and OE*Lae1* under saline conditions, relative to normal conditions (Supplementary Figure S10A,B). In this study, we observed that the length of the shoots and roots treated with OE*Vel1* and OE*Lae1* significantly increased relative to the wild type and control under both conditions (Supplementary Figure S10C,D). Similarly, the fresh weight of the roots or shoots of the maize plants treated with WT, OE*Vel1*, and OE*Lae1* under saline conditions was increased relative to the normal conditions (Supplementary Figure S10E,F). We observed a decrease in the fresh weight of plant roots and shoots treated with Δ*Vel1* and Δ*Lae1* under saline conditions relative to other treatments (Supplementary Figure S10E,F). The co-cultivation of maize with OE*Vel1* and OE*Lae1* decreased the accumulation Na^+^ in root as compared to the control (Supplementary Figure S10G). Knockout of *Vel1* and *Lae1* led to the accumulation of more Na^+^ in plants relative to the WT (Supplementary Figure S10G). These results specified that co-cultivation of maize and *T. asperellum* GDFS1009 decreases the accumulation of Na^+^ in the roots through the regulatory mechanisms of the *Vel1* and *Lae1* genes. There were no significant changes in the K^+^ content of WT, OE*Vel1*, and OE*Lae1* (Supplementary Figure S10H). The K^+^ content of the maize treated with *Vel1* and *Lae1* knockout was significantly reduced compared to the OE*Vel1* and OE*Lae1* (Supplementary Figure S10H).

## 4. Discussion

It is well documented that the use of plant growth-promoting and biocontrol fungus, *Trichoderma* spp., increases crop efficiency and decreases plant pathogens [22]. *Trichoderma* are broadly utilized to control plant diseases in different climatic zones [23], and their impact upon halo-tolerance has also been widely explored [24]. Various levels of salinity were found to differently affect the viability of *Trichoderma* and its efficacy upon plant growth. *Trichoderma* sense their environs by means of signal transduction pathways. Previously, the mitogen-activated protein kinase (MAPK) pathway was found to be involved in the halotolerance of *Trichoderma* [11]. The involvement of the velvet signal transduction pathway in several processes has been studied, such as *Trichoderma* growth, development, sporulation, secondary metabolite production, and stress tolerance [25]. Earlier studies illustrate that the velvet proteins interact with the putative methyltransferase and control the growth and development of *Trichoderma* through transcriptional regulation [26]. The role of *Vel1* and *Lae1* on the regulation of *Trichoderma* growth, survival, and tolerance under saline conditions has been not studied. Therefore, in this study, we examined the role of the *Vel1* and *Lae1* genes on salinity tolerance in free-living conditions and during co-cultivation with maize under saline circumstances.

*Trichoderma asperellum* responds well to the altered level of salinity. Its salinity tolerance was tested in three different media, including YM, PD, and VMS, in which NaCl concentrations has been varied to precisely define the tolerable level of NaCl concentrations. Notably, the growth of all strains was better in the YM and PD medium due to the higher quantity of nutrients. The growth of Δ*Vel1* and Δ*Lae1* was decreased in all three media, even under normal conditions, which revealed the importance of *Vel1* and *Lae1* to *Trichoderma* growth. This study revealed that the growth inhibition of *T. asperellum* is due to the Na^+^ content of the medium rather than the K^+^ content (Supplementary Figures S11 and S12), and further, this was supported by the inhibition of *T. asperellum* growth at 1 M NaCl. Our results indicated that the deletion of the *Vel1* and *Lae1* genes stopped the growth of *T. asperellum* under saline conditions (Figure 1A). This suggests the importance of the *Vel1* and *Lae1* genes to salinity tolerance.

In order to explore a promising relationship between the expression of genes involved in the oxidative stress response and salinity tolerance of *T. asperellum*, we recognized that NOX, SOD, and CAT enzymes and their gene expressions were increased in response to salinity. It has been observed that the knockout of the *Vel1* and *Lae1* genes reduced antioxidant activity and gene expression in order to make *T. asperellum* more susceptible to salinity. In the case of OE*Vel1* and OE*Lae1*, the NOX, CAT, and SOD were found to be upregulated under saline stress. This revealed that Vel1 and Lae1 proteins control the oxidative stress responsive genes globally upon ROS created by salinity. 

This study showed that glycerol was improved by saline stress. Glycerol was accumulated up to 34.25 ng^-g^/FW in the WT at 0.5 M NaCl. The concentration of glycerol was not only dependent upon the NaCl, but also upon regulatory genes such as MAPK and the Velvet complex. However, overexpression of *Vel1* and *Lae1* strains responded well to osmotic pressure by the increasing the accumulation of glycerol. Similar results have been observed in *Curvularia lunata* [27], i.e., the accumulation of glycerol during oxidative stress was reduced by the knockout of Cl*VelB*. The response of the *Vel1* and *Lae1* genes of *T. asperellum* to oxidative stress is in some ways similar to that of the *Vel1* genes of another fungus. Additionally, there was an increase in the regulation of genes associated with the synthesis of glycerol during saline stress. The 0.5 M NaCl in the medium triggered expression of the glycerol-3-phosphate dehydrogenase gene (GDP) in *T. asperellum* under osmotic stress. In several saline-tolerant fungi, the accumulation of glycerol is facilitated by the expression of GDP genes [28]. Similarly, our results also suggested the up-regulation of the GDP gene by the *Vel1* and *Lae1* genes.

Saline stress stimulates the MAPK signaling pathway. The best-known MAPK-signaling gene involved in saline stress is the HOG (high osmolarity of glycerol). Stimulation of the HOG gene encourages a higher glycerol content, strengthening of the cytoskeleton, cell wall dynamics, and osmolarity resistance [29]. Additionally, we found that expression of TMK2 and TMK3 was induced by the over-expression of the *Vel*1 and *Lae*1 genes in response to salinity. This confirms that the TMK2 and TMK3 increase under saline conditions was also co-regulated by the *Vel1* and *Lae1* genes. Further, TEM images confirmed the roles of *Vel1* and *Lae1* in cell wall integrity, namely through the strengthening the cell wall during the overexpression of the *Vel1* and *Lae1* genes under saline stress.

Proline is also assumed to be involved in several processes, such as the biosynthesis of protein, cell wall integrity maintenance, and ROS scavenging [30]. The greatest accumulation of proline protects the fungus from saline stress [31]. In the present study, proline accumulation was increased in *T. asperellum* under saline stress. High proline accumulation in *T. asperellum* leads to the maintenance of cell wall integrity and allows the *T. asperellum* to withstand saline stress and cell wall damage. The *Vel1* and *Lae1* genes proved that it is necessary to protect *T. asperellum* by increasing the accumulation of proline and thereby strengthening the cell wall integrity under saline stress.

In this investigation, the expression of five saline-tolerance genes from *T. asperellum* was examined during the growth of 0.5 M NaCl in response to *Vel1* and *Lae1*. During salinization, the expression of all five of these genes was induced by saline stress in the WT of *T. asperellum*. The most important Na^+^ efflux system is coded by ENA protein. Among them, ENA1 is the most vital protein for the maintenance of ion homeostasis and saline tolerance in fungi [20]. ENA1 acts like P-type Na^+^-ATPase, and it also facilitates Li^+^ or K^+^ efflux [32]. The regulation of the *ENA1* gene of fungus is controlled by the Na^+^ content of the environment [20,32]. The present investigation showed that the upregulation of the *ENA1* gene of *T. asperellum* at 0.5 M NaCl in the OE*Vel1* and OE*Lae1* strains. The results revealed the importance of *Vel1* and *Lae1* to the regulation of the *ENA1* gene and the subsequent maintenance of ion homeostasis.

In addition, Na^+^/H^+^ antiporters also played a significant part in balancing the intracellular H^+^ and Na^+^ homeostasis [33]. This antiporter exchanged the Na^+^ into the fungus and exported the H^+^ outside the fungus [34]. Na^+^/H^+^ antiporters are important for the Na^+^ and H^+^ homeostasis, saline tolerance, and the morphogenesis of fungus [35]. The gene expression study revealed that Na^+^/H^+^ antiporters were also induced by the *Vel1* and *Lae1* genes in response to NaCl. The cation H^+^ antiporter 1 (CPA1) was detected in *T. asperellum* CBS 433.97 using the reference whole genome sequence. This protein was categorized in order to catalyze the Na^+^:H^+^ exchange. The most important role of CPA1 is to regulate cytoplasmic pH by exporting the cytosol H^+^ (produced by the metabolism) outside the fungi and salt tolerance owing to Na^+^ uptake into vacuoles [36]. With the aim of discovering the role of the *T. asperellum* Ca^2+^ signaling mechanism in response to salinity tolerance, we examined the expression level of the sodium–calcium exchanger (NCX) gene. NCX protein has been reported only in *Aspergillus cristatus* [37]. The NCX protein is required to exchange sodium/calcium ions in fungus and thereby maintain Ca homeostasis [37]. Indeed, calcium signaling is required for regulation of the growth, sporulation, and survival of fungus in different environments. The expressions of NCX genes in *Aspergillus cristatus* were 8.94-fold higher under 3 M NaCl [37]. This suggests that NCX is required for salinity tolerance.

Proline is an important osmolyte which is increased in *T. asperellum* due to the influence of saline stress, and we studied the expression of the sodium/proline transporter (PutP) under saline conditions. PutP protein has a major role in the exchange of proline into the bacteria or fungus as an osmolyte to adapt to osmotic stress and maintain intracellular redox and ROS scavenging [38]. The expression of PutP in *T. asperllum* coincides with the results of the proline accumulation. This upregulation of PutP in response to salinity induces the accumulation of proline into the fungus in order to prevent cell wall damage created by osmotic stress. This investigation showed that the *Vel1* and *Lae1* genes regulate the saline-tolerant gene and the ion exchange mechanism under the osmotolerance of *T. asperellum*.

Under saline stress, thick cell walls were seen, which is due to the adaptation of *T. asperellum* to saline stress. Similar to the thickness of the cell wall, a rough surface over the cell wall was seen in *T. asperellum* subjected to 0.5 M NaCl. This rough surface was similar to the rough cell surface of *Epicoccum nigrum* [39]. The rough surface of the cell wall was due to the presence of exopolysaccharide. The formation of exopolysaccharide on the *Trichoderma* cell surface could help to decrease the permeability of the cell wall [40]. The cell wall thickness and exopolysaccharide were not increased in the strains of Δ*Vel1* and Δ*Lae1*. This provides evidence that the *Vel1* and *Lae1* genes also regulate the morphology of *T. asperllum* in order to survive under saline stress.

The *Vel1* and *Lae1* genes also played an important role in the maintenance of ionic homeostasis during saline tolerance. Conversely, the Vel1 and Lae1 proteins of *T. asperellum* increased the absorption of Na^+^ from the medium containing 0.5 M NaCl. However, these Vel1 and Lae1 proteins maintained the cytosolic Na^+^ content. Absorbed Na^+^ has been detected in the vacuoles of *T. asperellum* WT, OE*Vel1*, and OE*Lae1* but not in Δ*Vel1* and Δ*Lae1*. Vacuoles are the biggest subcellular organelle. They contribute in different ways to the maintenance of cellular homeostasis. The important role of the vacuole is to degrade cellular constituents, ionic and metabolic accumulation in order to maintain cellular homeostasis [41]. The vacuole is involved in the storage of the excess cations in the cytosol in order to regulate the growth and development of fungus [41]. In this study, for the first time, we proposed that Vel1 and Lae1 proteins regulate and control the other signaling pathways involved in ion homeostasis in order to accumulate Na^+^ ions inside the vacuole. TEM results showed that the number of vacuoles had been increased in the OE*Vel1* and OE*Lae1* strains compared to the WT grown under saline conditions, while the vacuoles were not clearly visualized in Δ*Vel1* and Δ*Lae1*. TEM EDS tests to assess Na^+^ ions in *T. asperellum* confirmed that the Na^+^ ions were accumulated in the vacuoles and not in the membrane or cytosol, or in other subcellular organelles such as the mitochondria and endoplasmic reticulum of OE*Vel1*, OE*Lae1*, and WT; meanwhile, a very low Na^+^ content was detected in the cell wall membrane of Δ*Vel1* and Δ*Lae1* but not in the vacuoles. Considering that the results of ionic transport gene expression and ionic localization clearly showed that the *Vel1* and *Lae1* genes form a complex and regulate other signal transduction genes to absorb the Na^+^ from the environment and accumulate it into the vacuoles, thereby contributing to the maintenance of the ionic homeostasis of *T. asperllum* and allowing it to grow and be physiologically active under saline conditions.

An additional feature discovered over the course of this work, which was mentioned earlier, is the capability of *T. asperellum* to mitigate saline for several plants [10,42,43]. In this investigation, OE*Vel1* and OE*Lae1* increased the branching and thickening of maize roots along with substantially reducing the Na^+^ concentration in the roots of maize grown under saline conditions (Supplementary Figure S13). The reduction of the Na^+^ concentration of the plant grown under saline conditions along with the *Trichoderma* revealed that *T. asperellum* prevented the absorption of Na^+^ into the roots [43]. The reduction of Na^+^ concentration in the roots of the plants grown under saline conditions might be due to the capability of *Trichoderma* to regulate plant genes either by reducing the concentration of Na^+^ absorption or by maintaining the ionic homeostasis. Earlier studies showed that the fungus grown under saline conditions activates the sodium, potassium transporter genes [20]. The outcomes of this study are also in accordance with the results of *Serendipita* [20], which protect the plants by absorbing Na^+^. In this circumstance, *Trichoderma* may absorb Na+, possibly into the vacuole, and eradicate it around the maize roots. Under several stress conditions, the Vel1 and Lae1 proteins of *Trichoderma* might perform different roles for the colonies in the plant roots. Perhaps, in the case of the *Trichoderma* exposed to saline stress, the Vel1 and Lae1 proteins may assist with the *T. asperellum* survival. In order to check the importance of *Vel1* and *Lae1* for plant protection against saline stress, the knockout and overexpression strains of the *Vel1* and *Lae1* genes were used to study the symbiotic association. The colonization ratio of the *Vel1* and *Lae1* knockout strains with the maize roots was reduced, and this revealed the importance of *Vel*1 and *Lae1* to the maize root colonization. It was further confirmed that *Vel1* and *Lae1* genes, during the colonization with maize roots, increased the salinity tolerance and mitigated the Na^+^ to the plants.

## 5. Conclusions

*Vel1* and *Lae1* together form a complex and coordinate with other factors in order to regulate the growth and development of *Trichoderma* under stress-response conditions. *Vel1* and *Lae1* strengthen the survival and growth of *T. asperellum* GDFS1009 in saline soil and water, thereby enhancing plant growth. Further, the Vel1 and Lae1 proteins are physiologically involved in the accumulation of osmolyte which maintains cell wall integrity and transport Na^+^ ions into the vacuoles in order to maintain cellular ion homeostasis through the regulation of genes involved in the MAPK signaling pathway and ionic membrane transport genes. Our results confirmed that *Vel1* and *Lae1* mitigate the absorption of Na^+^ content in maize roots during co-cultivation under saline conditions. This study proposed the importance of *Vel1* and *Lae1* against salinity and plant growth promotion for sustainable agriculture.

## Figures and Tables

**Table 1:** Maize root fungal colonization by *T. asperellum* WT, OE*Vel1*, OE*Lae1*, Δ*Vel1*, and Δ*Lae1* strains under normal and saline conditions in a hydroponics system.

Treatments	Plant		Fungi		Ratio ^c^
	Ct	Qty ^a^	Ct	Qty ^b^	
**Maize grown under normal condition**					
**Control**	19.44 ± 0.16	5.01 ± 0.04	-	-	-
**WT**	19.15 ± 0.3	5.09 ± 0.08	24.71 ± 0.2	5.75 ± 0.1	1.13 ± 0.04 ^b^
**OE *Vel***	18.64 ± 0.32	5.23 ± 0.08	23.04 ± 0.28	6.54 ± 0.13	1.25 ± 0.02 ^a^
**OE *Lae***	19.07 ± 0.30	5.11 ± 0.08	23.42 ± 0.26	6.37 ± 0.12	1.24 ± 0.02 ^a^
**D *Vel***	19.61 ± 0.28	4.96 ± 0.07	27.01 ± 0.25	4.66 ± 0.12	0.93 ± 0.02 ^c^
**D *Lae***	19.8 ± 0.26	4.91 ± 0.07	27.05 ± 0.27	4.64 ± 0.13	0.94 ± 0.04 ^c^
**Maize grown under saline condition**					
**Control**	21.74 ± 0.29	4.39 ± 0.08	-	-	-
**WT**	20.03 ± 0.23	4.85 ± 0.06	26.08 ± 0.31	5.10 ± 0.15	1.05 ± 0.01 ^b^
**OE *Vel***	19.27 ± 0.11	5.06 ± 0.03	24.79 ± 0.23	5.71 ± 0.11	1.13 ± 0.02 ^a^
**OE *Lae***	19.07 ± 0.18	5.13 ± 0.04	24.74 ± 0.29	5.73 ± 0.13	1.11 ± 0.03 ^a^
**D *Vel***	20.12 ± 0.37	4.83 ± 0.10	29.11 ± 0.28	3.65 ± 0.13	0.75 ± 0.02 ^c^
**D *Lae***	19.95 ± 0.46	4.87 ± 0.12	29.31 ± 0.55	3.56 ± 0.26	0.72 ± 0.03 ^c^

^a^ Quantity of plant DNA (ng) referred to actin gene. ^b^ Quantity of Trichoderma DNA (ng) referred to 18 s rRNA gene. ^c^ Ratio of fungal DNA vs. plant DNA. Values are the standard error means of three root samples from three independent experiments. Different lowercase letters indicate significant differences (*p* < 0.05) between the different treatments. -Absence of a Ct value.

## Data Availability

Not applicable.

## Supplementary Materials

The following supporting information can be downloaded at: https://www.mdpi.com/article/10.3390/jof8111176/s1, Table S1. Primers used in this study. Figure S1. Vel1 and Lae1 deletion strategy used by homologous recombination. Figure S2. Vector constructed for the overexpression of (A) Vel1 and (B) Lae1 gene. Figure S3. Confirmation of Vel1 and Lae1 gene deletion and overexpression. Lane 1- 1kb marker; Lane 2- ΔVel1 (absence of Vel1 gene); Lane 3- OEVel 1 (presence of Vel1 gene); Lane 4- OEVel 1 (presence of Vel1 gene along with promoter and terminator region of 1300th vector); Lane 5- ΔLae 1 (absence of Lae1 gene); Lane 6- OELae1 (presence of Lae1 gene); Lane 7- OELae 1 (presence of Lae1 gene along with promoter and terminator region of 1300th vector). Figure S4. The relative expression of Vel1 and Lae1 gene under (E) normal and (F) saline condition as the fold ratio between the WT grown under normal conditions. Figure S5. The impact of Vel1 and Lae1 on the growth of T. asperellum GDFS1009. Figure S6. Growth of T. asperellum WT, OEVel1, OELae1, ΔVel1 and ΔLae1 in both normal and saline conditions at three different media. (A) Growth of T. asperellum WT and mutants were studied in PD medium supplemented with 250 mM, 500 mM, 1 M and 1.5 M NaCl. (B) Growth of T. asperellum WT and mutants were studied in YM medium supplemented with 250mM, 500mM, 1M and 1.5M NaCl. (C) Growth of T. asperellum WT and mutants were studied in VMS medium supplemented with 250mM, 500mM, 1M and 1.5M NaCl. Values are the average of biological triplicates. Error bars represent the standard error. Bars with different letters represent a statistically significant difference from each other at the level of *p* < 0.05 based on the ANOVA. Figure S7. PCA analysis on the growth of T. asperellum WT, OEVel1, OELae1, ΔVel1 and ΔLae1 in both normal and saline conditions at three different media. (A) Growth of T. asperellum WT and mutants were studied in PD medium supplemented with 250 mM, 500 mM, 1 M and 1.5 M NaCl. (B) Growth of T. asperellum WT and mutants were studied in YM medium supplemented with 250 mM, 500 mM, 1 M and 1.5 M NaCl. (C) Growth of T. asperellum WT and mutants were studied in VMS medium supplemented with 250 mM, 500 mM, 1 M and 1.5 M NaCl. Figure S8. Growth and survival of T. asperellum WT, OEVel1, OELae1, ΔVel1 and ΔLae1 in the (A) normal soil, (B) normal water, (C) saline soil and (D) saline water. Values are the average of biological triplicates. Error bars represent the standard error. Boxes with different letters represent a statistically significant difference from each other at the level of *p* < 0.05 based on the ANOVA. Figure S9. microscopic images of maize root fungal colonization by T. asperellum WT, OEVel1, OELae1, ΔVel1 and ΔLae1 strains under normal and saline conditions in hydroponics system. Figure S10. Influence of T. asperellum WT, OEVel1, OELae1, ΔVel1 and ΔLae1 strains on the growth and Na+ mitigation of maize seedlings. Growth of maize under normal (A) and saline (B) conditions in hydroponics system. Shoot length (C), root length (D), shoot weight (E), root weight (F), sodium (G) and potassium (H) content under normal and saline conditions. Values are the average of five biological replicates. Error bars represent the standard error. Bars with different letters represent a statistically significant difference from each other at the level of *p* < 0.05 based on the ANOVA. Figure S11. Growth of T. asperellum WT, OEVel1, OELae1, ΔVel1 and ΔLae1 in both normal and saline conditions at three different media. (A) Growth of T. asperellum WT and mutants were studied in PD medium supplemented with 250 mM, 500 mM, 1 M and 1.5 M KCl. (B) Growth of T. asperellum WT and mutants were studied in YM medium supplemented with 250 mM, 500 mM, 1 M and 1.5 M KCl. (C) Growth of T. asperellum WT and mutants were studied in VMS medium supplemented with 250 mM, 500 mM, 1 M and 1.5 M KCl. Figure S12. Growth of T. asperellum WT, OEVel1, OELae1, ΔVel1 and ΔLae1 in (A) YM Broth (B) YM Broth supplemented with 0.5 M NaCl and (C) YM Broth supplemented with 0.5 M KCl. Figure S13. sporulation of T. asperellum WT, OEVel1, OELae1, ΔVel1 and ΔLae1 in (A) YM Broth and (B) YM Broth supplemented with 0.5 M NaCl.
