# Supplementary material for: Role of the Global Fitness Regulator Genes on the Osmotic Tolerance Ability and Salinity Hazard Alleviation of Trichoderma asperellum GDFS 1009 for Sustainable Agriculture
Source: J Fungi (Basel). 2022 Nov 8;8(11):1176. doi: 10.3390/jof8111176 (PMC9698259; doi:10.3390/jof8111176)
Supplement: Supplementary file 1 [file jof-08-01176-s001.zip › jof-2007134-supplementary.pdf]

Journal of Fungi

MDPI

Table S1. Primers used in this study.

Primer	Sequence	Comments
**Up Vel1-F**	GGCATTTTGCTGCTTGTACC	Deletion of Vel1 in *T. asperellum* GDFS1009
**Up Vel1-R**	GTTCACTGTGGCTGCGATAA	
**Up Vel1 Hind III F**	CCCAAGCTTGGCATTTTGCTGCTTGTACC	
**Up Vel1 sal I R**	ACGCGTCGACGTTCACTGTGGCTGCGATAA	
**Dn Vel1-F**	CTCGGCTCCGATGTATGAGT	
**Dn Vel1-R**	ACCGGCCACTATTGTTGAAT	
**Vel DN sac1 -F**	GGTGGTCGAGCTCGACCGGCCACTATTGTTGAAT	
**VEL dn Bam h1- R**	GTCGCGGGATCCCTCGGCTCCGATGTATGAGT	
**PtrpC F**	GGGAAGCTTGGAGGTCAACACATCAATGC	Overexpression of Vel1 in *T. asperellum* GDFS1009
**PtrpC R**	ACACTCGAAGGTGTCGCCATTTGGATGCTTGGGTAGAATA	
**CDS F**	TATTCTACCCAAGCATCCAAATGGCGACACCTTCGAGTGT	
**CDS R**	TGATTTCAGTAACGTTAAGTTTATACCTGGTACTGGTTGA	
**TtrpC F**	TCAACCAGTACCAGGTATAAACTTAACGTTACTGAAATCA	
**TtrpC R**	GGTCGACAACCCAGGGGCTGGTGACGG	
**Lae1 up F**	GCGAAATATCCCCGTGTAAG	Deletion of Lae1 in *T. asperellum* GDFS1009
**Lae1 up R**	GGAAATAGGAATCCGGGAAA	
**Lae up Hind F**	CCcAAGCTTGGGTTGGCGAAAAAATTCC	
**Lae up kPN R**	ACTCTCAATTTTCAAACGGGGTACCCCG	
**Lae1 dn F**	ACGGGTTAAAGCCGAAACTT	
**Lae1 dn R**	TCTCCTCCTCCCGGTCTACT	
**Lae DN BAM F**	GATCCGCGACGACTGCGTTCTGCG	
**Lae DN XBA R**	GGGAGGAGAAAGAGAACTAGTCTAGACTAG	
**Lae1 orf f**	ATGTCGTCTCGAAACGCT	Overexpression of Lae1 in *T. asperellum* GDFS1009
**Lae1 orf R**	TTACAGAGGTCTACAACATCCTC	
**LAE-Pro-F**	ACGACGGCCAGTGCCAAGCTTGGAGGTCAACACATCAATGCC	
**LAE-Pro-R**	GCCCATGATGACATTTGGATGCTTGGGTAGAATAGGTAA	
**LAE-F**	ATGTCATCATGGGCATCAAGC	
**LAE-R**	GAAACTCGAGTCAGATGCTGCTCCGCCTTCGCCCAC	
**LAE-T-F**	TCTGACTCGAGTTTCTCCATAATAAT	
**LAE-T-R**	GATGTGTTGACCTCCGAATTCAACTTAATAACACATTGCGGACG	
**Vel1F**	TCTCGACCGACCTACTGAGG	Transcriptional quantification of Vel1 gene
**Vel1R**	GAGGGCTCCAGGTCAAAGTC	
**Lae1F**	ACAGCATCCCTCCAGGTGTA	Transcriptional quantification of Lae1 gene
**Lae1R**	GAGCAGCCGAAGATGGATCA	
**ENA1 F1**	TGACACTCTTTCCCGTGCTC	Transcriptional quantification of ENA1 gene
**ENA1 R1**	GTTTGCACCACTTCCACGTC	
**NHE F1**	TTATGAGCTCAAGGCTCGGC	Transcriptional quantification of NHE gene
**NHE R1**	GGGAGTGCACACTGCAAAAG	
**CPA1 F1**	GTCGTCGTGGTCTTGACTGT	Transcriptional quantification of CPA1 gene
**CPA1 R1**	ATGTCAAACTCGTCGTCGCT	
**NCX F1**	CGATGAGATCGATGCAGGCT	Transcriptional quantification of NCH gene
**NCX R1**	GTCGACGATGTTGATTGCCG	
**PutP F1**	TGCAATCCCCTGGTGCTTAG	Transcriptional quantification of PutP gene
**PutP R1**	GGCATTACCAGCCCGTTAGA	
**TMK2 F**	CCAGCCCGACCATCATGTCTC	Transcriptional quantification of TMK2 gene
**TMK2 R**	CGCATAATCTCTTGGTAAATCAGTTG	
**TMK3 F**	GCA GGT CGG TTC CGA GAA GC	Transcriptional quantification of TMK3 gene
**TMK3 r**	GCA GGT CGG TTC CGA GAA GC	
**NOX F**	CACCACCTGTTCATCCC	Transcriptional quantification of NOX gene
**NOX R**	GTCAAATGGCGAGAATCC	
**CAT F**	ACTGCATTGTCCGTTTCT	Transcriptional quantification of CAT gene
**CAT R**	AGTTGCCCTCCTCTGTG	
**SOD -F**	GATGCTCAGGGTAACGCCAA	Transcriptional quantification of SOD gene
**SOD -R**	TTGCCAGTCTTGAGGGACTC	
***18S rRNA* F**	GGTGGAGTGATTTGTCTG	Transcriptional quantification of 18S rRNA gene and internal reference gene
***18S rRNA* R**	CTTACTAGGGATTCCTCG	
**ACTIN F**	CTCTCAGCACATTCCAGCAG	Transcriptional quantification of ACTIN gene of Maize
**ACTIN F**	AGGAGGACGGCGATAACAG	

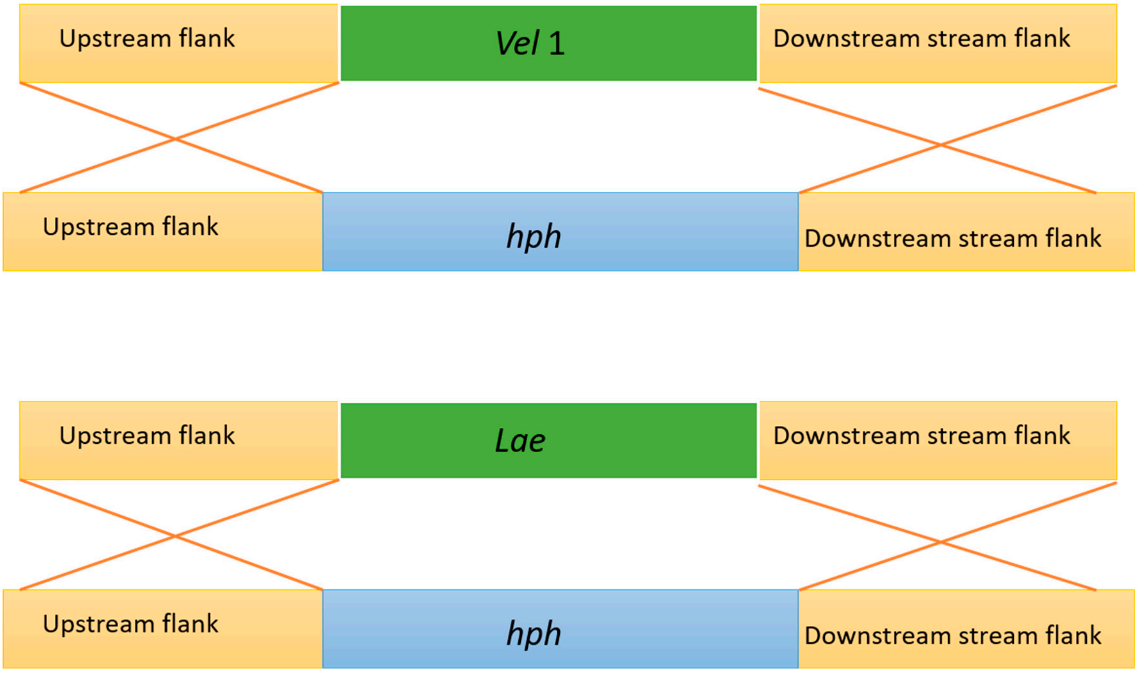

Figure S1. *Vel1* and *Lae1* deletion strategy used by homologous recombination.

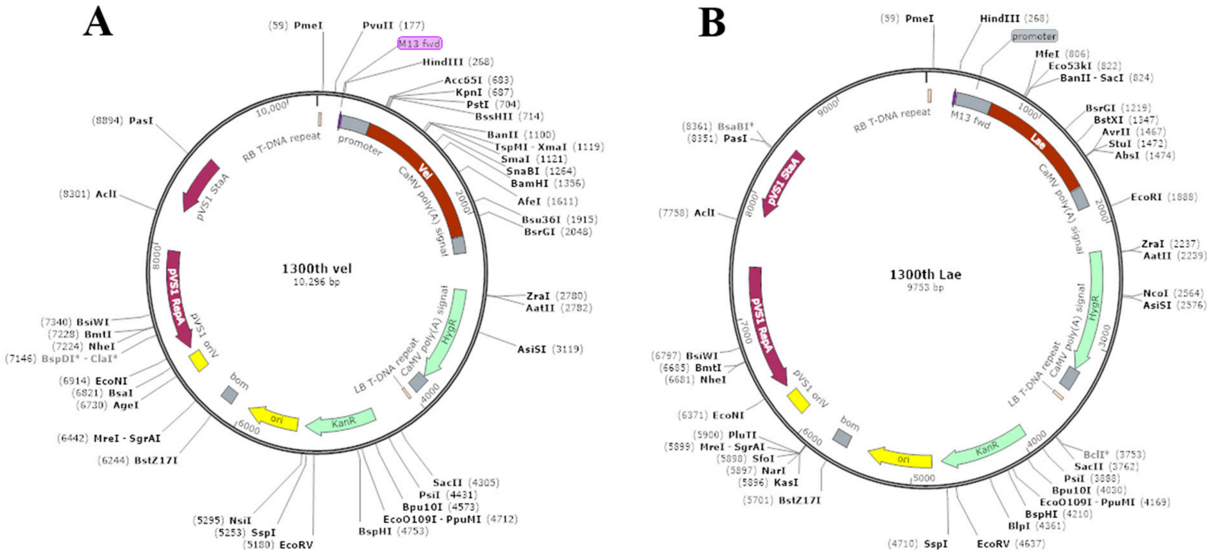

Figure S2. Vector constructed for the overexpression of **(A)** *Vel1* and **(B)** *Lae1* gene.

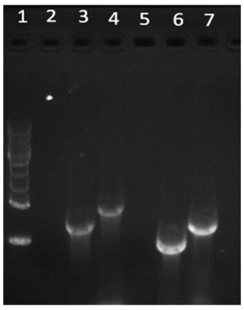

Figure S3. Confirmation of *Vel1* and *Lae1* gene deletion and overexpression. Lane 1- 1kb marker; Lane 2- ΔVel1 (absence of *Vel1* gene); Lane 3- OEVel 1 (presence of *Vel1* gene); Lane 4- OEVel 1 (presence of *Vel1* gene along with promoter and terminator region of 1300th vector); Lane 5- ΔLae 1

(absence of *Lae1* gene); Lane 6- OELae1 (presence of *Lae1* gene); Lane 7- OELae 1 (presence of *Lae1* gene along with promoter and terminator region of 1300th vector).

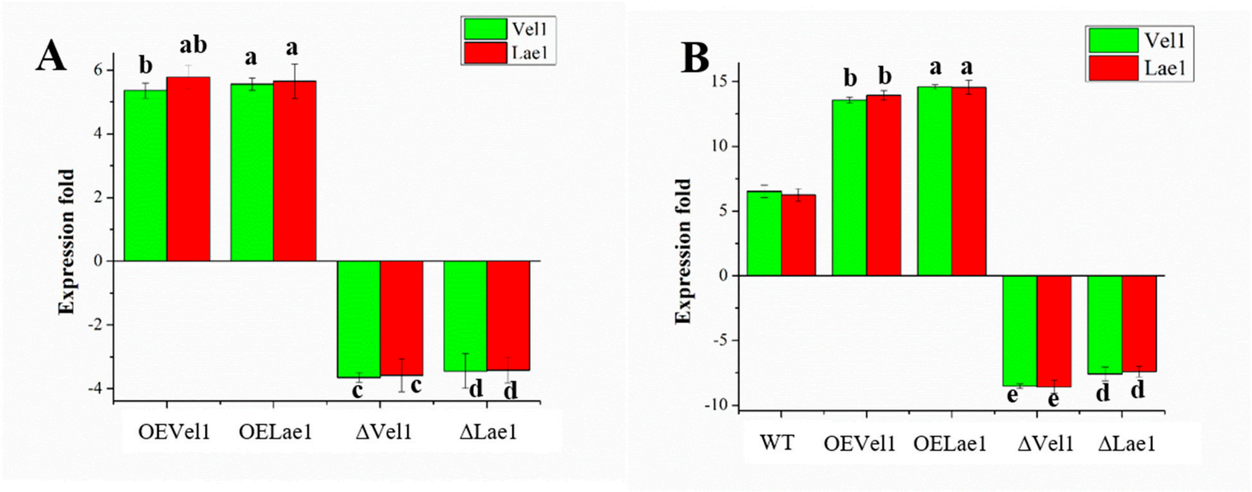

Figure S4. The relative expression of *Vel1* and *Lae1* gene under **(A)** normal and **(B)** saline condition as the fold ratio between the WT grown under normal conditions.

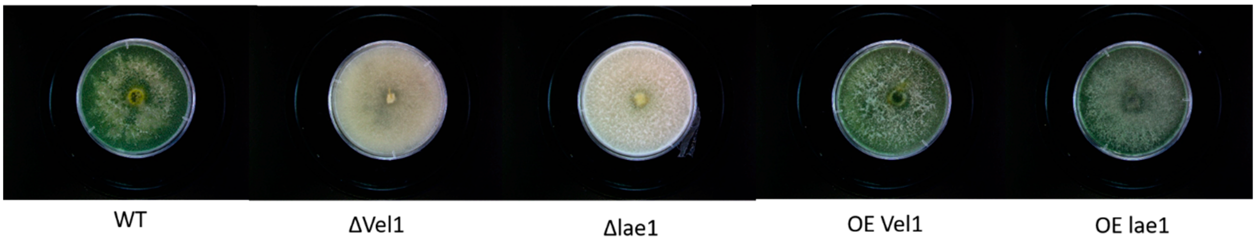

Figure S5. The impact of *Vel1* and *Lae1* on the growth of *T. asperellum* GDFS1009.

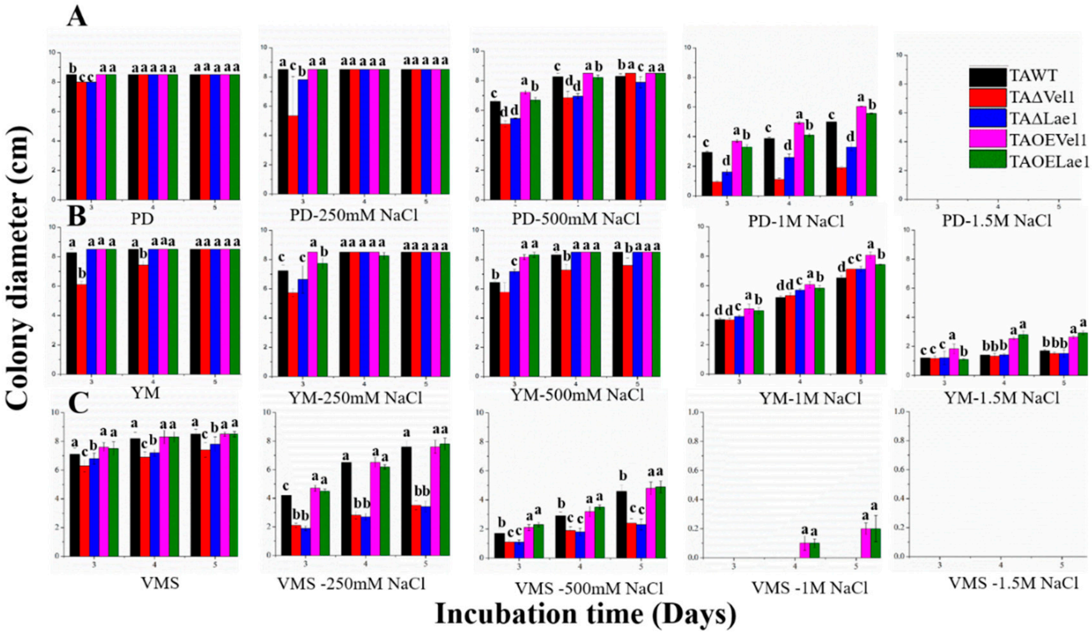

Figure S6. Growth of *T. asperellum* WT, OE*Vel*1, OE*Lae*1, Δ*Vel*1 and Δ*Lae*1 in both normal and saline conditions at three different media. **(A)** Growth of *T. asperellum* WT and mutants were studied in PD medium supplemented with 250 mM, 500 mM, 1 M and 1.5 M NaCl. **(B)** Growth of *T. asperellum* WT and mutants were studied in YM medium supplemented with 250mM, 500mM, 1M and 1.5M

NaCl. **(C)** Growth of *T. asperellum* WT and mutants were studied in VMS medium supplemented with 250mM, 500mM, 1M and 1.5M NaCl. Values are the average of biological triplicates. Error bars represent the standard error. Bars with different letters represent a statistically significant difference from each other at the level of $P < 0.05$ based on the ANOVA.

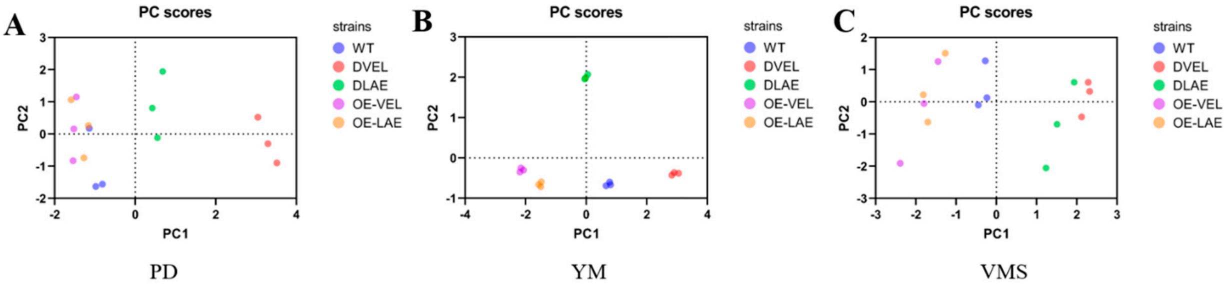

Figure S7. PCA analysis on the growth of *T. asperellum* WT, OE*Vel1*, OE*Lae1*, Δ*Vel1* and Δ*Lae1* in both normal and saline conditions at three different media. **(A)** Growth of *T. asperellum* WT and mutants were studied in PD medium supplemented with 250 mM, 500 mM, 1 M and 1.5 M NaCl. **(B)** Growth of *T. asperellum* WT and mutants were studied in YM medium supplemented with 250 mM, 500 mM, 1 M and 1.5 M NaCl. **(C)** Growth of *T. asperellum* WT and mutants were studied in VMS medium supplemented with 250 mM, 500 mM, 1 M and 1.5 M NaCl.

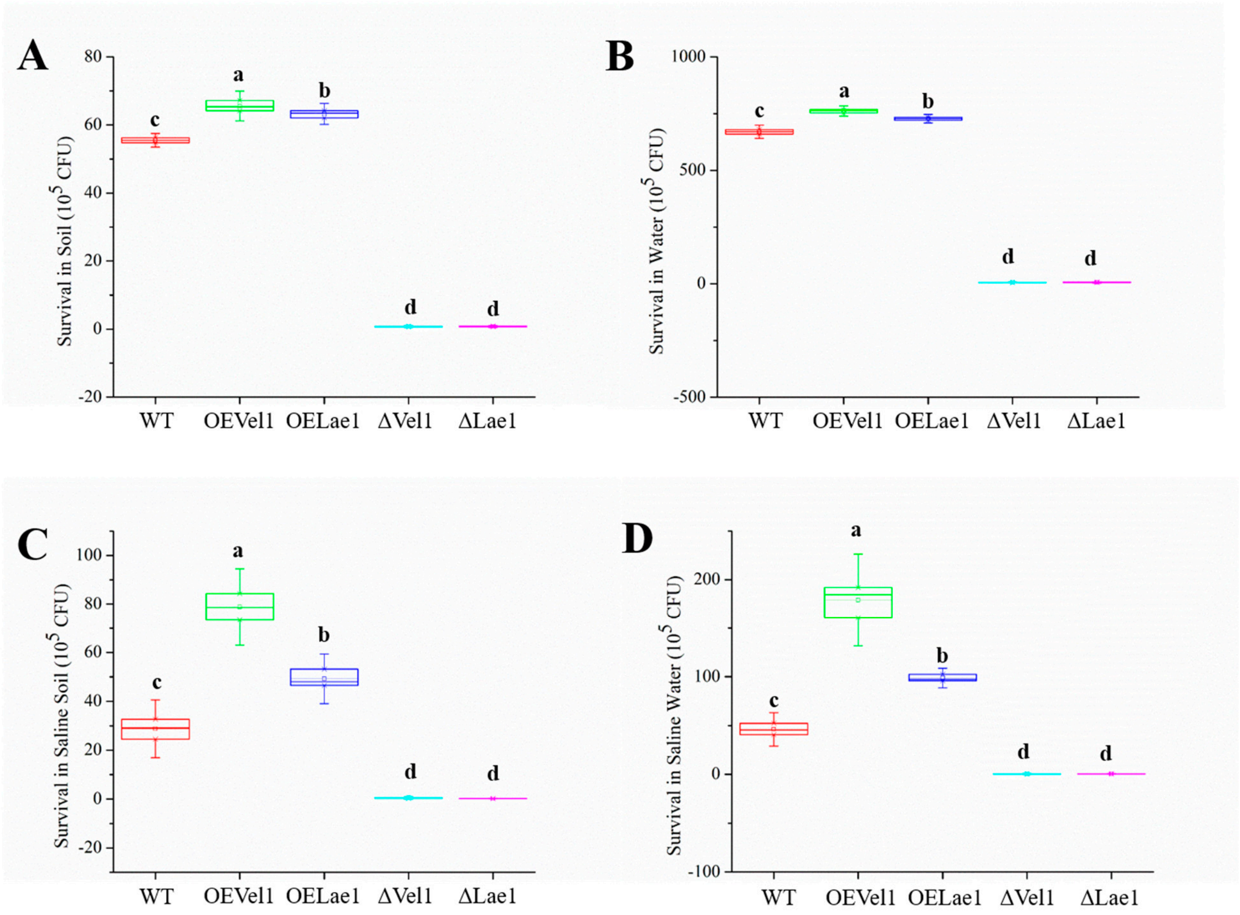

Figure S8. Growth and survival of *T. asperellum* WT, OE*Vel*1, OE*Lae*1, Δ*Vel*1 and Δ*Lae*1 in the **(A)** normal soil, **(B)** normal water, **(C)** saline soil and **(D)** saline water. Values are the average of biological triplicates. Error bars represent the standard error. Boxes with different letters represent a statistically significant difference from each other at the level of $P < 0.05$ based on the ANOVA.

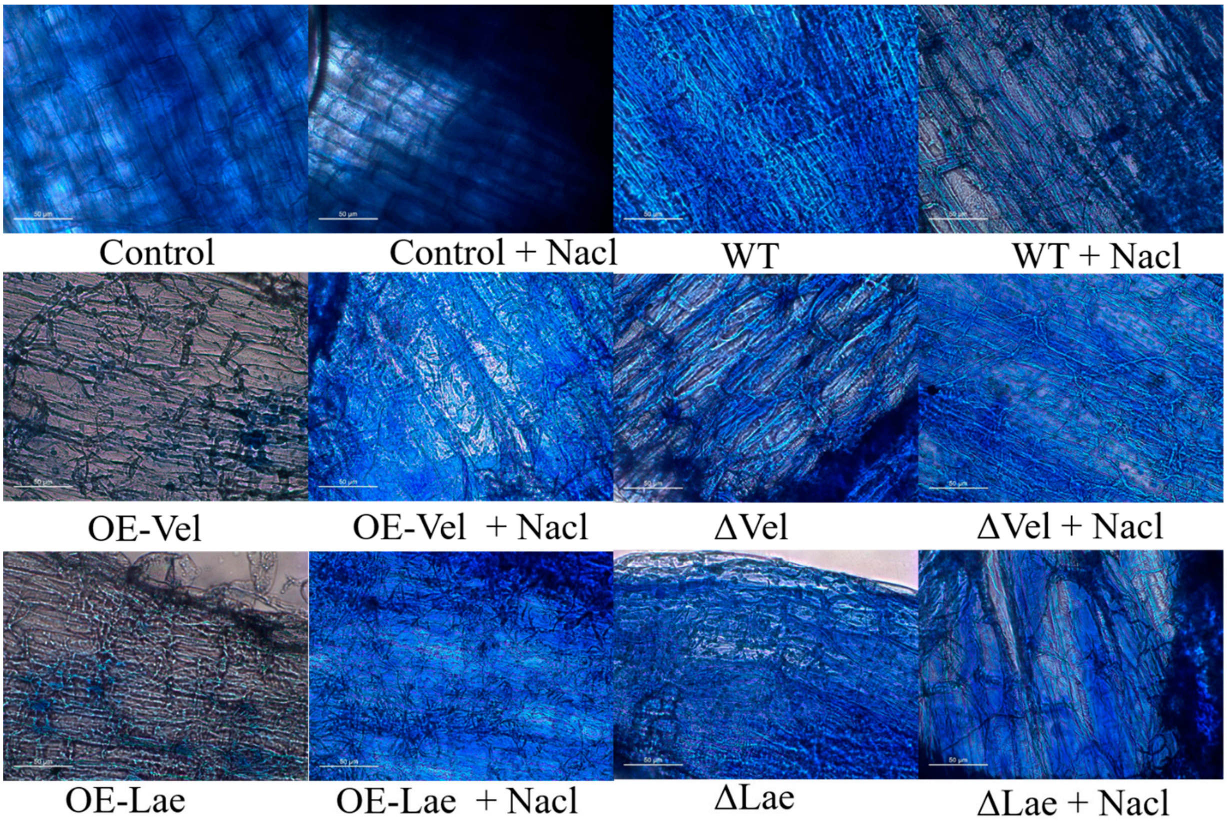

Figure S9. microscopic images of maize root fungal colonization by *T. asperellum* WT, OE*Vel1*, OE-*Lae1*, Δ*Vel1* and Δ*Lae1* strains under normal and saline conditions in hydroponics system.

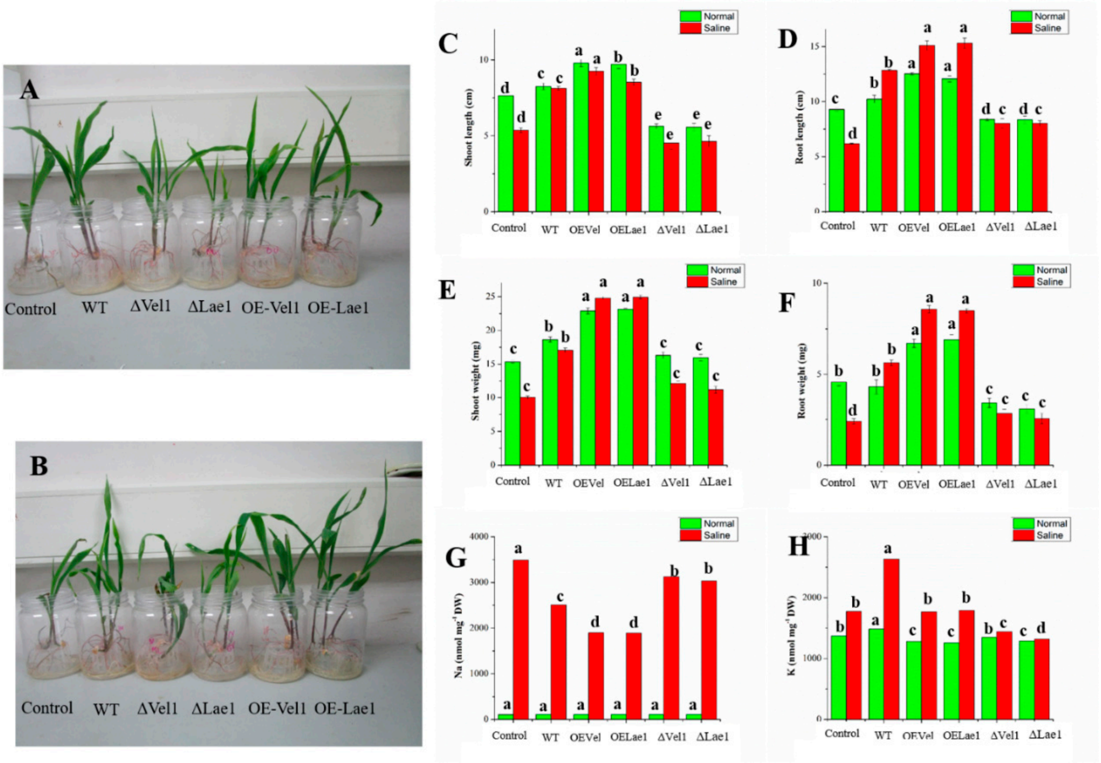

Figure S10. Influence of *T. asperellum* WT, OE*Vel*1, OE*Lae*1, Δ*Vel*1 and Δ*Lae*1 strains on the growth and Na+ mitigation of maize seedlings. Growth of maize under normal **(A)** and saline **(B)** conditions in hydroponics system. Shoot length **(C)**, root length **(D)**, shoot weight **(E)**, root weight **(F)**,

sodium **(G)** and potassium **(H)** content under normal and saline conditions. Values are the average of five biological replicates. Error bars represent the standard error. Bars with different letters represent a statistically significant difference from each other at the level of $P < 0.05$ based on the ANOVA.

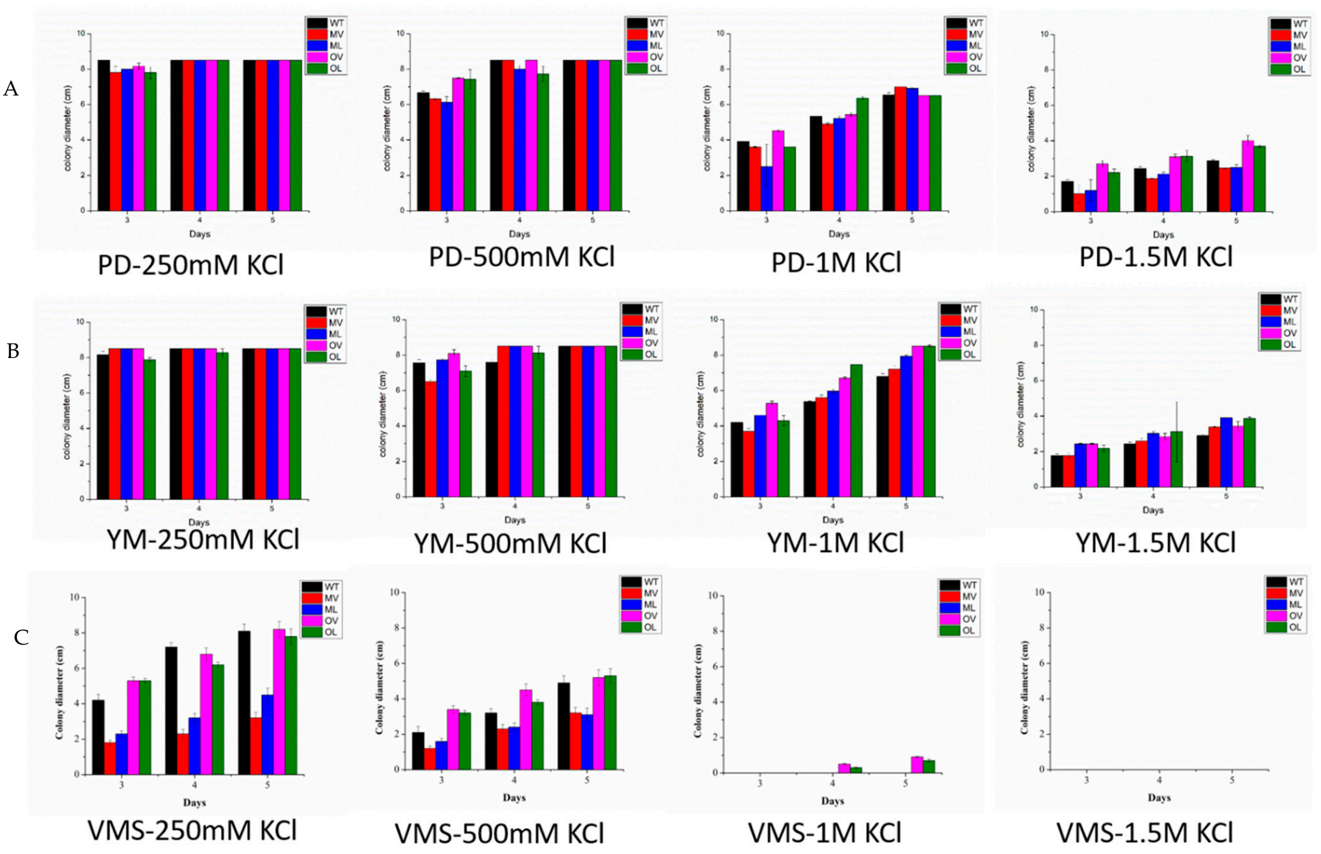

Figure S11. Growth of *T. asperellum* WT, OE*Vel1*, OE*Lae1*, Δ*Vel1* and Δ*Lae1* in both normal and saline conditions at three different media. **(A)** Growth of *T. asperellum* WT and mutants were studied in PD medium supplemented with 250 mM, 500 mM, 1 M and 1.5 M KCl. **(B)** Growth of *T. asperellum* WT and mutants were studied in YM medium supplemented with 250 mM, 500 mM, 1 M and 1.5 M KCl. **(C)** Growth of *T. asperellum* WT and mutants were studied in VMS medium supplemented with 250 mM, 500 mM, 1 M and 1.5 M KCl.

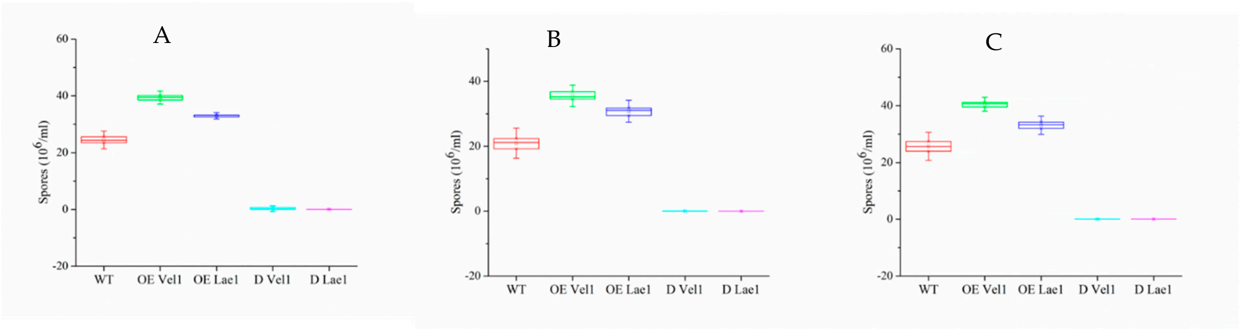

Figure S12. Growth of *T. asperellum* WT, OE*Vel1*, OE*Lae1*, Δ*Vel1* and Δ*Lae1* in **(A)** YM Broth **(B)** YM Broth supplemented with 0.5 M NaCl and **(C)** YM Broth supplemented with 0.5 M KCl.

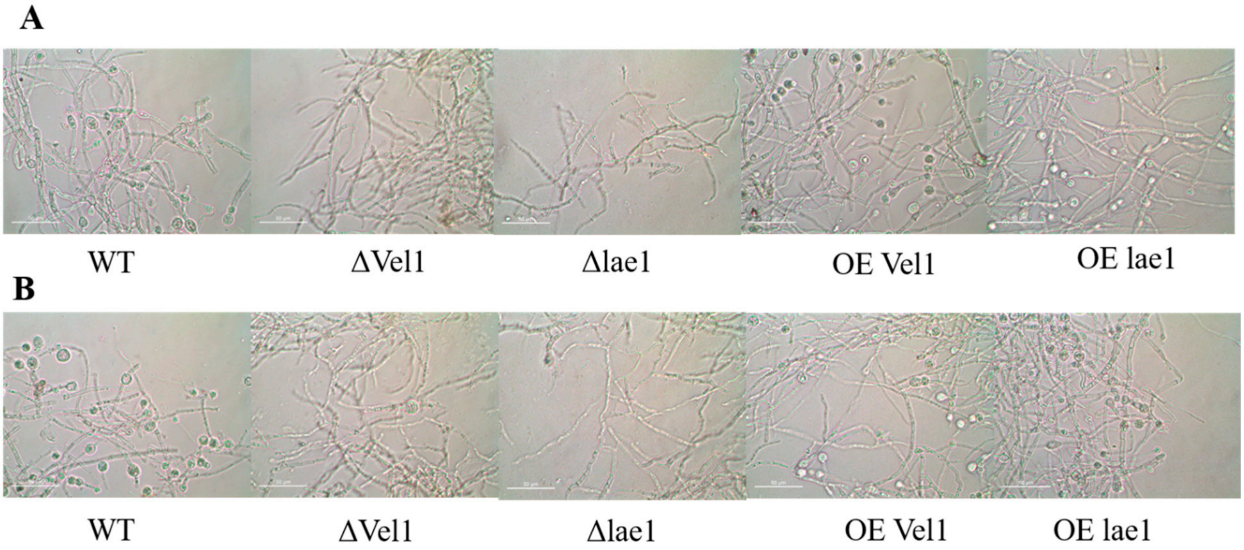

Figure S13. sporulation of *T. asperellum* WT, OE*Vel1*, OE*Lae1*, Δ*Vel1* and Δ*Lae1* in **(A)** YM Broth and **(B)** YM Broth supplemented with 0.5 M NaCl.
